# Observations on the Management of the Poor in Scotland, and Its Effects on the Health of the Great Towns

**Published:** 1840-04

**Authors:** 


Art. XV.—*Observations on the Management of the Poor in Scotland, and its Effects on the Health of the Great Towns.* By W. P. Alison, m.d., f.r.s.e.—*Edinburgh,* 1840. 8vo, pp. 198.

We hope soon to have an opportunity of treating at large of the subject of this small but most valuable work; we cannot, however, allow the present quarter to pass without expressing our sense of its vast importance, and offering our tribute of praise and admiration to the patriotic and benevolent physician who has called the attention of his countrymen to it, in a tone which cannot be misunderstood, and will hardly, we think, be resisted. The object of Dr. Alison is to prove the necessity of extending the provisions of the English Poor Law, or a modification of it, to Scotland,—a necessity founded on the melancholy fact (too fully proved in his pages) of the total insufficiency of private or non-compulsory charity to supply the poor in Scotland with food and clothing adequate to the prevention of disease and death to a frightful extent.

"In following out this enquiry I have long since formed," says Dr. Alison, "and do not scruple to express an opinion, which I cannot expect to be in the first instance either well received or generally credited in this country, viz., that the higher ranks in Scotland do much less (and what they do less systematically, and therefore less effectually,) for the relief of poverty and of sufferings resulting from it than those of any other country in Europe which is really well regulated; and much less than experience shows to be necessary in any long-inhabited and fully-peopled country, in order that the lower ranks may be maintained in tolerable comfort, and a proper foundation laid for their religious and moral improvement." (*Preface,* p. viii.)

The natural result is, that in Edinburgh and Glasgow "the annual proportion of deaths to the population is not only *much beyond the average of Britain, but very considerably greater than that of London!*"

We earnestly recommend this book to the attention of our readers both in England and Scotland, and beg they will lose no opportunity of naming it and its terrible subject to all their friends in the legislature of this country. It cannot fail, sooner or later, to lead to a legislative enactment to cure the evils depicted in it;—an enactment which will hallow the name of Dr. Alison to the poor of all Scotland for ever, as it is already hallowed to the poor of his native city by the devotion and services of half a lifetime.

